# Characteristics of an ideal nebulized antibiotic for the treatment of pneumonia in the intubated patient

**DOI:** 10.1186/s13613-016-0140-x

**Published:** 2016-04-18

**Authors:** Matteo Bassetti, Charles-Edouard Luyt, David P. Nicolau, Jérôme Pugin

**Affiliations:** Infectious Diseases Clinic, Santa Maria Misericordia University Hospital, Udine, Italy; Service de Réanimation, Institut de Cardiologie, Groupe Hospitalier Pitié–Salpêtrière, Assistance Publique–Hôpitaux de Paris, Paris, France; Sorbonne Universités, UPMC Université Paris 06, INSERM, UMRS_1166-ICAN Institute of Cardiometabolism and Nutrition, Paris, France; Center for Anti-Infective Research and Development, Hartford Hospital, Hartford, USA; Service des Soins Intensifs, University Hospitals of Geneva and Faculty of Medicine, University of Geneva, Geneva, Switzerland

**Keywords:** Hospital-acquired pneumonia, Ventilator-associated pneumonia, Gram-negative bacteria, Multidrug resistance, Systemic antibiotics, Nebulizers, Inhaled antibiotics, Clinical cure

## Abstract

Gram-negative pneumonia in patients who are intubated and mechanically ventilated is associated with increased morbidity and mortality as well as higher healthcare costs compared with those who do not have the disease. Intravenous antibiotics are currently the standard of care for pneumonia; however, increasing rates of multidrug resistance and limited penetration of some classes of antimicrobials into the lungs reduce the effectiveness of this treatment option, and current clinical cure rates are variable, while recurrence rates remain high. Inhaled antibiotics may have the potential to improve outcomes in this patient population, but their use is currently restricted by a lack of specifically formulated solutions for inhalation and a limited number of devices designed for the nebulization of antibiotics. In this article, we review the challenges clinicians face in the treatment of pneumonia and discuss the characteristics that would constitute an ideal inhaled drug/device combination. We also review inhaled antibiotic options currently in development for the treatment of pneumonia in patients who are intubated and mechanically ventilated.

## Background

### Management of pneumonia in the intensive care unit (ICU) remains challenging

**Fig. 1:**
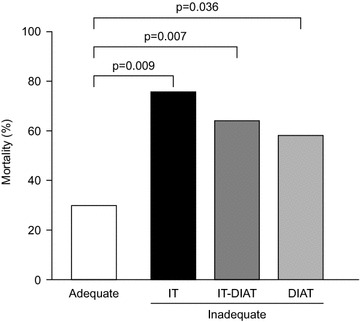
Mortality rates observed in patients with ventilator-associated pneumonia who received adequate, inadequate (IT-DIAT inadequate), inappropriate therapy (IT) or delayed initiation of appropriate therapy (DIAT). Adapted from Ref. [5]. Figure reproduced with permission from the European Respiratory Society who are the copyright holders for this material

Hospital-acquired pneumonia (HAP) and ventilator-associated pneumonia (VAP) remain important causes of morbidity and mortality despite advances in antimicrobial therapy [1]. Patients with severe pneumonia or critical illness often require intubation and mechanical ventilation to manage acute respiratory failure; furthermore, 9–27 % of intubated patients will develop VAP [1, 2]. In mechanically ventilated patients with VAP, attributable mortality estimates vary considerably and have been reported to range from 0 to 50 % [3, 4]; however, there are large differences between subgroups of patients, and VAP-attributable mortality may be as high as 69 % in surgical patients for example [3]. Failure to provide timely and effective therapy in the first 48 h is also linked to particularly high mortality (Fig. 1) [5]. Clearly, early initiation of appropriate antibiotics is essential for effective management.

Current treatments are typically given through the intravenous (IV) route; however, despite widespread implementation of current antibiotic guidelines for the treatment of pneumonia, clinical cure rates rarely exceed 60 %, and recurrence rates remain high [6–10]. The high prevalence of multidrug-resistant (MDR) pathogens such as the ‘ESKAPE’ species (in particular *Staphylococcus aureus, Klebsiella pneumoniae, Acinetobacter baumannii, Pseudomonas aeruginosa* and *Enterobacter* spp.) add to the increasing difficulty of treating VAP. Unsurprisingly, MDR pathogens are associated with significant attributable mortality [11], and their increasing prevalence has been a concern due to the limited number of new antibiotics currently in development [12, 13]; clearly, there is an urgent clinical need to optimize therapy for critically ill patients with pneumonia [14].

## What makes antibiotics effective at the site of infection?

Effective treatment of bacterial pneumonia requires the concentration of the antibiotic in the lung to exceed the minimum inhibitory concentration (MIC) of the infecting pathogen. However, while some antimicrobial drugs such as fluoroquinolones penetrate well into lung tissue when administered intravenously [15], others (e.g., β-lactams, colistin, aminoglycosides and glycopeptides such as vancomycin) have poor lung penetration and tissue distribution [16–18]. Poor lung penetration of drugs can be overcome by dose increases, but this management approach is often limited by the associated risk of systemic adverse events; for example, high systemic concentrations of aminoglycosides are associated with nephrotoxicity and ototoxicity [19, 20]. The effectiveness of IV antibiotic therapy may be further diminished by pharmacokinetic changes in critically ill patients, including changes in absorption, distribution and elimination [21]. Such patient-specific variation makes adequate dosing of antibiotics challenging and may result in the delivery of drug concentrations that are either too low (and therefore sub-therapeutic) or too high (and therefore toxic) [22, 23].

In mechanically ventilated and intubated patients with pneumonia, targeting antibiotics to the lungs via aerosolization could offer a way to achieve high exposures of antibiotics directly at the site of infection, while minimizing systemic side effects [24, 25]. Initial treatment with aerosolized antibiotics combined with IV therapy is therefore a promising treatment strategy that could improve clinical outcomes.

## Potential benefits of nebulized antibiotics

Previous approaches to nebulized antibiotic therapy have had several clinical and technical limitations, including sub-optimal delivery and lack of drugs specifically formulated for aerosolization [26, 27]. While issues such as these have hampered aerosolized delivery techniques, several recent developments suggest that these shortcomings could soon be eliminated.

### Nebulized antibiotics can achieve high drug concentrations in the lung

Perhaps the key advantage of administering antibiotics by inhalation rather than via IV infusion is the potential to deliver high concentrations of antibiotic directly to the site of lung infection [28, 29]. Animal studies in ventilated piglets have demonstrated that nebulized antibiotics achieved high deposition in infected lung parenchyma with concentrations far above the MICs for most Gram-negative strains [30], and indeed, the efficiency of bacterial killing in piglets inoculated with *E. coli* was greater after nebulization compared with intravenous administration [31]. Furthermore, clinical studies have shown that inhaled tobramycin, for example, can achieve high bronchial concentrations, and inhaled amikacin can reach epithelial lining fluid (ELF) concentrations far in excess of the MICs for Gram-negative strains usually responsible for pneumonia [32, 33]. These concentrations may also exceed the MICs for MDR pathogens.

### Nebulized antibiotics are associated with low systemic exposure

The high lung concentrations achieved with inhaled antibiotics are paired with low systemic absorption [34]; indeed, administering antibiotics such as aminoglycosides by aerosolization generates significantly lower peak serum concentrations compared with intravenous administration [27, 35]. One potential benefit of lower systemic concentrations is a reduced incidence of adverse events, such as nephrotoxicity [27]. In addition, low systemic concentrations may also have the benefit of falling outside the mutant selection window, thus reducing the risk of systemic resistance development [36, 37]. Studies in patients with cystic fibrosis treated with aerosolized antibiotics have not reported an increase in the emergence of resistance with inhaled therapy compared with standard therapy or placebo [38, 39]. This is supported by a recent double-blind placebo-controlled study of patients in the ICU, which demonstrated that in comparison with placebo, aerosolized antibiotics were not associated with the development of new antibiotic resistance [40].

### Inhaled administration may reduce the need for systemic antibiotics

Aerosolized antibiotic therapy also provides the potential for a reduction in the overall use of systemic antibiotics [24, 41], with clear benefits for antibiotic stewardship and management of emergent resistance. Increased resistance due to frequent or excessive use of systemic antimicrobials has been documented for several drug classes. For example, between 2007 and 2011 the increased use of amoxicillin/clavulanic acid, ceftazidime and cefepime, carbapenems, and fluoroquinolones has correlated with an increase in the incidence of resistance in isolates of *K. pneumoniae*, *P. aeruginosa* and AmpC-producing *Enterobacteriaceae*, among others [42, 43]; decreasing the general use of antibiotics is therefore a key aim of antimicrobial stewardship programs. Importantly, this goal may be aided by the wider use of aerosolized therapies, and results from a Phase II study demonstrated that inhaled antibiotics could significantly reduce the use of IV antibiotics [44].

## Characteristics of an ‘ideal’ inhaled antibiotic: what needs to be optimized?

Improving outcomes for critically ill patients with pneumonia requires the optimization of clinical factors such as ventilator settings, device usability and patient safety. An ideal inhaled antibiotic therapy should have a suitable formulation for aerosolization and consistently deliver high antibiotic concentrations to the site of infection via an efficient device; the drug should also have limited systemic penetration to prevent unwanted side effects.

### Formulation of the ideal antibiotic for aerosolization

**Fig. 2:**
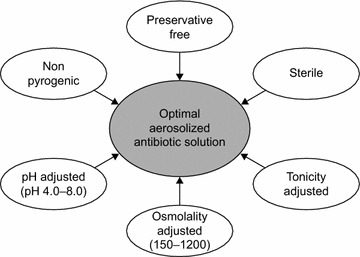
Ideal properties of an antibiotic solution for aerosolization. Adapted from information in references [26, 27, 29]

Currently available IV drug formulations are not optimized for aerosolization and often have properties that may impede drug delivery [26]. In addition, IV formulations usually contain preservatives such as phenols and many have sub-optimal osmolarity (<150 mOsm/L, >1200 mOsm/L), which can increase bronchospasm and coughing [26, 27]. To be suitable for aerosolization, the formulation should be sterile, preservative-free and non-pyrogenic. It should also be adjusted for the lung environment with a suitable pH (4.0–8.0), osmolarity (150–1200 mOsm/L) and tonicity [26, 27, 29] (Fig. 2). A solution that is specifically formulated for inhalation could minimize adverse effects, such as airway irritation, and increase delivery efficiency. Currently, the only antimicrobials that have a specific formulation developed for inhalation are colistin [45], aztreonam [46] and tobramycin [47], all of which are approved exclusively for use in cystic fibrosis [48].

### Optimizing the dose

The choice of dose in early studies assessing aerosol delivery of antibiotics was sometimes based on the packaging of parenteral IV antibiotics, rather than on an a priori definition of the amount of drug that was needed in the lower airway [49]. Since then, studies have generally selected doses designed to achieve lung concentrations that far exceed the MIC of relevant pathogens in the lung [41, 49]. One recent study, for example, assessed two different dosing regimens of an inhaled aminoglycoside against a stringent pharmacokinetic target of achieving 25 times the highest MICs reported for *P.aeruginosa* and *Acinetobacter* spp. in North American ICUs [41].

### Characteristics of the ideal delivery device

**Fig. 3:**
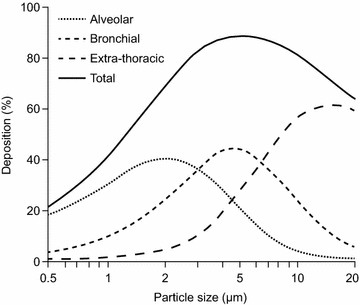
Relationship between aerodynamic diameter and site of lung deposition. The optimal particle size for deposition throughout the lung (total) is 3–5 µm. Adapted from Ref. [70]. Figure reproduced with permission, the publisher for this copyrighted material is Mary Ann Libert, Inc. publishers

For optimal therapeutic effect, an appropriately formulated antibiotic should be used in combination with an efficient delivery device. A recent meta-analysis of inhaled treatments showed that while nebulized antibiotics (with or without IV antibiotic) may improve clinical cure rates compared with IV antibiotics alone, nebulizers themselves vary considerably in efficiency [50]. Indeed, jet nebulizers are known to have considerably lower efficiency (i.e., drug delivery rates) than vibrating mesh nebulizers (<15 vs 40–60 %, respectively) [51]. However, even within the vibrating mesh nebulizer device category, there is significant variation in delivery efficiency (Table 1). One of the primary determinants of delivery efficiency and drug deposition is particle size; a consistent and optimal particle size promotes distribution throughout the lungs and avoids condensation (‘rain out’) within the ventilator circuit [27, 52]. Particle size is usually measured as mass median aerodynamic diameter (MMAD) or volumetric median diameter (VMD), and a particle size in the range of 1–5 µm is considered to be suitable for deposition in the lung [36]; within this range, an MMAD or VMD of 3–5 µm is considered optimal for deposition in the bronchial conducting airways and throughout the alveoli (Fig. 3). However, currently available jet nebulizers cannot produce such small particles [52].

**Table 1:** Technical considerations and performance characteristics of vibrating mesh nebulizers

Technical consideration	Performance characteristics vibrating mesh nebulizers		
	Bayer Amikacin Inhale	Aerogen Aeroneb Solo	PARI eFlow
Mode of action	Breath synchronized (hand-held = continuous nebulization)	Continuous nebulization	Breath enhanced
Delivered dose	On-vent: 35–58 %; hand-held: 35–64 % [54]	13–17 % [71, 72]	31–44 % [73]
Delivery time	Timing depends on patient and flow rate but has been reported to be 36 ± 16 (on-vent) and 15 ± 5 (hand-held) [54]	Dependent on medication but suggested to be around 7–10 min with 3 mL albuterol [71, 72]	Dependent on medication [63]
Humidification	Humidification does not affect delivered dose of amikacin Recommended to remove HME device	Recommended to turn humidification off during delivery Remove HME as per manufacturer’s instructions	Humidification can be left on during delivery [66]

New drug–device combinations may hold promise in this area. The pulmonary drug delivery system (PDDS) device currently in development (NKTR-061) is an adaptive vibrating mesh nebulizer which has been combined with a specially formulated Amikacin Inhalation Solution (BAY41-6551). With this combination, approximately 60 % of the inhaled dose is delivered to the lung [53, 54]. These data indicate that specifically designed drug–device combinations have the potential to consistently generate and deliver optimally sized drug particles homogeneously throughout the lung, including to the peripheral airways. Any new drug–device combination that can deliver particles within the optimal 3–5 µm range could facilitate high drug concentrations at the site of pneumonia infection [36, 55].

### Ease of use: ventilator setting adjustments

A key practical consideration for an ideal nebulizer is its ease of use in the hospital setting. For intubated and mechanically ventilated patients, the nebulizer should integrate directly into the ventilator circuit, with only minimal need to adjust ventilator settings or remove humidification [27, 52]. However, current-generation nebulizers, regardless of the drug formulation used, require careful attention to be paid to ventilator settings (e.g., choice of ventilator mode—pressure versus volume-controlled, inspiratory time, inspiratory flow, tidal volume, duty cycle and respiratory rate) to ensure optimal performance [53] and may also necessitate increased sedation of the patient as these settings are altered. Such adjustments can be complex due to the wide variety of modes and settings available, and guidelines advise avoiding heavy sedation if possible [1]. These complexities currently make the use of nebulization technically demanding; in order to optimize this route of administration, standardized aerosolization procedures are required [35]. The increased sedation of patients to enable better synchronization to ventilator settings may also be associated with longer durations of mechanical ventilation; one study reported no statistically significant difference in the duration of mechanical ventilation between patients treated with aerosolized antimicrobials compared with those treated with intravenous antimicrobials [35]. If a nebulizer device could be added to the circuit without the need for ventilator setting adjustment, this could minimize delays in treatment and reduce undesirable increases in sedation.

Synchronizing aerosol generation with the inspiratory flow of the ventilator could also enhance drug delivery [56]. An in vivo study found that levels of antibiotic delivered with breath-synchronized nebulization were four to seven times higher than with continuous nebulization, depending on the extent of ventilator humidification [57]. While humidification improves patient outcomes and prevents adverse events such as hypothermia, bronchospasm and cilia damage, studies (primarily conducted with jet nebulizers or metered-dose inhalers) have demonstrated that humidification greatly reduces drug delivery efficiency with conventional inhaled treatment approaches [53, 58]. Therefore, clinicians currently have to choose between the removal of humidification (thus improving delivery efficiency but potentially exposing the patient to adverse side effects) or the retention of humidification (protecting the patient but compromising delivery efficiency). An ideal inhaled antibiotic should therefore achieve consistent lung delivery irrespective of humidification.

## Overview of current understanding of inhaled treatments in intubated patients

The use of nebulized antibiotics has been increasing steadily since the 1970s, and inhaled antibiotic therapy is being revisited as a potential treatment option due to the surge in pneumonia caused by MDR bacteria [36]. Aerosolized therapy offers a way to administer high doses of antibiotics that exert their efficacy against pathogens directly at the site of infection while reducing systemic toxicity.

### Evidence from clinical trials

Inhaled colistin has shown clinical utility in pneumonia, generating high lung concentrations and achieving efficient bacterial killing with a clinical cure rate of approximately 67 % [25, 51, 59]. Other studies have further demonstrated that nebulized antibiotics, such as specially formulated Amikacin Inhale, produce low systemic concentrations with limited toxic side effects [33], and a recent meta-analysis supports that nebulized antibiotics may improve clinical cure rates in patients with VAP, although additional clinical studies are still needed [50].

Indeed, despite some promising data, the overall number of well-designed trials examining the efficacy and tolerability of nebulized antibiotics remains low. Many studies have only involved a single center and are confounded by inadequate patient enrollment, poor methodology or failures in standardizing or reporting delivery methods and particle sizes [52]. Currently, there is no standardized technique for administration of a given aerosolized drug, and different studies have used different doses or formulations as well as differing patient cohorts. These factors make the comparison of efficiency and tolerability data difficult and pose challenges when trying to standardize this method of treatment and decide best practice. There is a clear unmet need for further multicenter studies, with standardized methodologies, consistent dosing and larger cohorts to improve the data available on the efficacy and safety of inhaled therapy.

### Treatment guidelines

Aerosolized adjunctive therapy with IV antimicrobials is suggested by the ATS/IDSA guidelines (2005) as appropriate for patients with pneumonia caused by MDR Gram-negative organisms who are not responding to systemic therapy alone; however, these guidelines are now a decade old and may no longer be an accurate reflection of the bacterial landscape currently confronting clinicians in the ICU. Other more recent guidelines, such as the Canadian HAP/VAP guideline (2008), recommend the use of aerosolized vancomycin in patients infected with MRSA; other guidelines currently make no recommendations for the use of inhaled antibiotics [60, 61].

### Healthcare worker perceptions and understanding

While there is now a more positive attitude toward nebulized antibiotics, clinician knowledge pertaining to the delivery of aerosolized therapy remains poor. Techniques to improve nebulizer output, such as reducing the inspiratory flow, are not regularly employed, and potentially dangerous practices (such as connecting the nebulizer to an external gas source or never changing the expiratory limb filter) were reported in a recent survey into physicians’ practice, knowledge and beliefs regarding aerosol therapy [62].

## Inhaled therapies currently in development

Aerosolized antibiotics have been used off-label for the treatment of pneumonia in mechanically ventilated, critically ill patients for around 40 years, but there is still no consensus, guideline or FDA-approved product available for such treatment [24]. There are, however, three aerosol-based therapies currently in development for the treatment of pneumonia in this vulnerable patient group.

The PARI eFlow rapid nebuliser system^®^ [63] is a multiple-use, single-patient device that is placed on the inspiratory limb of the ventilator circuit [52] to deliver a combination of amikacin and fosfomycin; the two drugs are added sequentially to the nebulizer. In two Phase I studies, the PARI system generated small amikacin–fosfomycin droplets and achieved high tracheal aspirate concentrations with low systemic exposure [64–66]. When delivered using a drug ratio of 5:2 (amikacin–fosfomycin), the combination reduced resistance development, and the authors attributed this effect to synergy between the two antibiotics [64–66]. A Phase II trial with the PARI system is ongoing (NCT01969799).

Inhaled tobramycin (TOBI^®^) is approved by the FDA for use in patients with cystic fibrosis and is currently under investigation for use in patients with pneumonia. Early studies have suggested that TOBI^®^ is well tolerated and effective for the treatment of VAP caused by *P. aeruginosa* or *Acinetobacter* spp., with reduced systemic side effects compared with IV tobramycin [67]. While these results are promising, the study was performed using a very small cohort, with only five patients in each group.

Amikacin Inhale, a drug–device combination being developed to treat patients who are intubated and mechanically ventilated who develop Gram-negative pneumonia, consists of a specifically formulated Amikacin Inhalation Solution and the pulmonary drug delivery system (PDDS) (NKTR-061, BAY41-6551). The PDDS synchronizes aerosolization of the antibiotic with the first 75 % of the inspiratory flow, with the aim of enhancing deposition in the lung and reducing wastage. The system can also be used in on-vent and hand-held configurations in a number of orientations to allow therapy to continue after extubation, with no need for ventilator setting or dose adjustment [54, 68]. In an in vitro study, the on-vent configuration of the PDDS (and Amikacin Inhalation Solution) achieved an estimated lung dose (ELD) of 35–58 % of the nominal dose, while the hand-held configuration achieved an ELD of 35–64 % of the nominal dose [54]. Phase II studies have demonstrated that the concentration of amikacin delivered to the lung substantially exceeded MIC values for the Gram-negative organisms primarily responsible for pneumonia (NCT01004445 and NCT01021436) [33, 41]. In the first study, the median ELF amikacin concentration, as determined by bronchoalveolar lavage, was 976.1 µg/mL [33]. In the second study, the primary endpoint of achieving both a tracheal aspirate amikacin concentration ≥6,400 µg/mL (25 times the reference MIC of 256 µg/mL) and a ratio of amikacin aspirate AUC_0–24h_ to MIC ≥ 100 at day 1 was achieved in 50 % of patients receiving Amikacin Inhale 400 mg every 12 h [41]. Amikacin Inhale can achieve consistently high drug delivery independent of humidity or humidification method [69]. Maximum concentrations of amikacin in serum remained below the recommended maximal trough concentration for systemic amikacin administration, and Amikacin Inhale was generally well tolerated [41]. Two Phase III studies are ongoing (NCT01799993 and NCT00805168).

## Conclusions

Nebulizer technology continues to evolve. Improvements in nebulizer capabilities may offer new treatment options that maximize the potential benefits of inhaled antibiotic therapy. Recent advances are promising to deliver nebulized therapy options with optimal particle sizes and to achieve improved drug delivery throughout the lung, while maintaining low systemic exposures. The combination of specifically designed drug formulations and modern, high efficiency delivery devices has the potential to overcome current challenges in the aerosolized treatment of pneumonia [29].

Increasing resistance and limited efficacy of currently available IV antibiotics for the treatment of pneumonia in intubated and mechanically ventilated patients are a growing cause for concern, and the choice of effective treatments is limited. New therapy options are urgently needed; continued improvements in antibiotic formulations and nebulizer system designs provide an increasingly positive outlook for the future of inhaled antibiotics.

## Abbreviations

ATS: American Thoracic Society
CAP: community-acquired pneumonia
ELD: estimated lung dose
ELF: epithelial lining fluid
FDA: food and drug administration
HAP: hospital-acquired pneumonia
ICU: intensive care unit
IDSA: Infectious Diseases Society of America
IV: intravenous
MDR: multidrug resistant
MIC: minimum inhibitory concentration
MMAD: mass median aerodynamic diameter
MRSA: methicillin-resistant staphylococcus aureus
PDDS: pulmonary drug delivery system
VAP: ventilator-associated pneumonia
VMD: volumetric median diameter

## References

[1] American Thoracic Society/Infectious Diseases Society of America (ATS/IDSA) (2005). Guidelines for the management of adults with hospital-acquired, ventilator-associated, and healthcare-associated pneumonia. Am J Respir Crit Care Med.

[2] Kalanuria AA, Zai W, Mirski M (2014). Ventilator-associated pneumonia in the ICU. Crit Care.

[3] Melsen WG, Rovers MM, Groenwold RHH, Bergmans DCJJ, Camus C, Bauer TT, Hanisch EW, Klarin B, Koeman M, Krueger WA, Lacherade JC, Lorente L, Memish ZA, Morrow LE, Nardi G, van Nieuwenhoven CA CA, O’Keefe GE, George Nakos G, Scannapieco FA, Seguin P, Staudinger T, Topeli A, Ferrer M, Bonten MJM (2013). Attributable mortality of ventilator-associated pneumonia: a meta-analysis of individual patient data from randomised prevention studies. Lancet Infect Dis.

[4] Koenig SM, Truwit JD (2006). Ventilator-associated pneumonia: diagnosis, treatment, and prevention. Clin Microbiol Rev.

[5] Luna C, Aruj P, Niederman M, Garzón J, Violi D, Prignoni A, Ríos F, Baquero S, Gando S (2006). Appropriateness and delay to initiate therapy in ventilator-associated pneumonia. Eur Respir J.

[6] Chastre J, Wunderink R, Prokocimer P, Lee M, Kaniga K, Friedland I (2008). Efficacy and safety of intravenous infusion of doripenem versus imipenem in ventilator-associated pneumonia: a multicenter, randomized study. Crit Care Med.

[7] Jenkins S, Fisher A, Peterson J, Nicholson S, Kaniga K (2009). Meta-analysis of doripenem vs comparators in patients with Pseudomonas infections enrolled in four phase III efficacy and safety clinical trials. Curr Med Res Opin.

[8] Kollef M, Chastre J, Clavel M, Restrepo M, Michiels B, Kaniga K, Cirillo I, Kimko H, Redman R (2012). A randomized trial of 7-day doripenem versus 10-day imipenem-cilastatin for ventilator-associated pneumonia. Crit Care.

[9] Awad SS, Rodriguez AH, Chuang YC, Marjanek Z, Pareigis AJ, Reis G, Scheeren TWL, Sánchez AS, Zhou X, Saulay M, Engelhardt M (2014). A phase 3 randomized double-blind comparison of ceftobiprole medocaril versus ceftazidime plus linezolid for the treatment of hospital-acquired pneumonia. Clin Infect Dis.

[10] Flume P, VanDevanter D (2015). Clinical applications of pulmonary delivery of antibiotics. Adv Drug Deliv Rev.

[11] Bercault N, Boulain T (2001). Mortality rate attributable to ventilator-associated nosocomial pneumonia in an adult intensive care unit: a prospective case–control study. Respir Ther..

[12] Boucher H, Talbot G, Bradley J, Edwards J, Gilbert D, Rice L, Scheld M, Spellberg B, Bartlett J (2009). Bad bugs, no drugs: no ESKAPE! An update from the Infectious Diseases Society of America. Clin Infect Dis.

[13] Spellberg B, Bartlett J, Wunderink R, Gilbert DN (2015). Novel approaches are needed to develop tomorrow’s antibacterial therapies. Am J Respir Crit Care Med.

[14] Bassetti M, De Waele JJ, Eggimann P, Garnacho-Montero J, Kahlmeter G, Menichetti F, Nicolau DP, Paiva JA, Tumbarello M, Welte T, Wilcox M, Zahar RJ, Poulakou G (2015). Preventive and therapeutic strategies in critically ill patients with highly resistant bacteria. Intensive Care Med.

[15] Wise R, Honeybourne D (1999). Pharmacokinetics and pharmacodynamics of fluoroquinolones in the respiratory tract. Eur Respir J.

[16] Honeybourne D (1994). Antibiotic penetration into lung tissues. Thorax.

[17] Cruciana M, Gatti G, Lazzarini L, Furlan G, Broccali G, Malena M, Franchini C, Concia E (1996). Penetration of vancomycin into human lung tissue. J Antimicrob Chemother.

[18] Imberti R, Cusato M, Villani P, Carnevale L, Iotti G, Langer M, Regazzi M (2010). Steady-state pharmacokinetics and BAL concentration of colistin in critically ill patients after IV colistin methanesulfonate administration. Chest.

[19] Avent ML, Rogers BA, Cheng AC, Paterson DL (2011). Current use of aminoglycosides: indications, pharmacokinetics and monitoring for toxicity. Int Med J.

[20] Arnold A, Brouse S, Pitcher W, Hall R (2010). Empiric therapy for Gram-negative pathogens in nosocomial and health care-associated pneumonia: starting with the end in mind. J Intensive Care Med.

[21] Smith B, Yogaratnam D, Levasseur-Franklin K, Forni A, Fong J (2012). Introduction to drug pharmacokinetics in the critically ill patient. Chest.

[22] Blot S, Pea F, Lipman J (2014). The effect of pathophysiology on pharmacokinetics in the critically ill patient—concepts appraised by the example of antimicrobial agents. Adv Drug Deliv Rev.

[23] Vinks A, Derendorf H, Mouton J (2014). Fundamentals of Antimicrobial Pharmacokinetics and Pharmacodynamics.

[24] Palmer LB (2011). Aerosolized antibiotics in the intensive care unit. Clinics Chest Med.

[25] Lu Q, Luo R, Bodin L, Yang J, Zahr N, Aubry A, Golmard J, Rouby J (2012). Efficacy of high-dose nebulized colistin in ventilator-associated pneumonia caused by multidrug-resistant *Pseudomonas aeruginosa* and *Acinetobacter baumannii*. Anesthesiology.

[26] Le J, Neuhauser M, Brown J, Gentry C, Klepser M, Marr A, Schiller D, Schwiesow J, Tice S, VandenBussche H, Wood C (2010). Consensus summary of aerosolized antimicrobial agents: application of guideline criteria. Pharmacotherapy.

[27] Wood C (2011). Aerosolized antibiotics for treating hospital-acquired and ventilator-associated pneumonia. Exp Rev Anti-Infect Ther.

[28] Luyt C-E, Combes A, Nieszkowska A, Trouillet J, Chastre J (2009). Aerosolized antibiotics to treat ventilator-associated pneumonia. Curr Opin Infect Dis.

[29] Abu-Salah T, Dhand R (2011). Inhaled antibiotic therapy for ventilator-associated tracheobronchitis and ventilator-associated pneumonia: an update. Adv Ther.

[30] Goldstein I, Wallet F, Robert J, Bequemin M, Marquette C, Rouby J (2002). Lung tissue concentrations of nebulized amikacin during mechanical ventilation in piglets with healthy lungs. Am J Respir Crit Care Med.

[31] Goldstein I, Wallet F, Nicolas-Robin A, Ferrari F, Marquette C-H, Rouby J-J, the Experimental Intensive Care Unit Study Group (2002). Lung deposition and efficiency of nebulized amikacin during *Escherichia coli* pneumonia in ventilated piglets. Am J Respir Crit Care Med.

[32] Badia J, Soy D, Adrover M, Ferrer M, Sarasa M, Alarcón A, Codina C, Torres A (2004). Disposition of instilled versus nebulized tobramycin and imipenem in ventilated intensive care unit (ICU) patients. J Antimicrob Chemother.

[33] Luyt C-E, Clavel M, Guntupalli K, Johannigman J, Kennedy J, Wood C, Corkery K, Gribben D, Chastre J (2009). Pharmacokinetics and lung delivery of PDDS-aerosolized amikacin (NKTR-061) in intubated and mechanically ventilated patients with nosocomial pneumonia. Crit Care.

[34] Cooney G, Lum B, Tomaselli M, Fiel S (1994). Absolute bioavailability and absorption characteristics of aerosolized tobramycin in adults with cystic fibrosis. Clin Pharmacol.

[35] Lu Q, Yang J, Liu Z, Gutierrez C, Aymard G, Rouby JJ, the Nebulized Antibiotics Study Group (2011). Nebulized ceftazidime and amikacin in ventilator-associated pneumonia caused by *Pseudomonas aeruginosa*. Am J Respir Crit Care Med.

[36] Dhand R (2007). The role of aerosolized antimicrobials in the treatment of ventilator-associated pneumonia. Respir Care.

[37] Drlica K, Zhao X (2007). Mutant selection window hypothesis updated. Clin Infect Dis.

[38] Ramsey BW, Dorkin HL, Eisenberg JD, Gibson RL, Harwood IR, Kravitz RM, Schidlow DV, Wilmott RW, Astley SJ, McBurnie MA, Wentz K, Smith AL (1993). Efficacy of aerosolized tobramycin in patients with cystic fibrosis. N Engl J Med.

[39] Burns JL, Van Dalfsen JM, Shawar RM, Otto KL, Garber RL, Quan JM, Montgomery AB, Albers GM, Ramsey BW, Smith AL (1999). Effect of chronic intermittent administration of inhaled tobramycin on respiratory microbial flora in patients with cystic fibrosis. J Infect Dis.

[40] Palmer LB, Smaldone GC (2014). Reduction of bacterial resistance with inhaled antibiotics in the intensive care unit. Am J Respir Crit Care Med.

[41] Niederman M, Chastre J, Corkery K, Fink J, Luyt C-E, Sanchez Garcia M (2012). BAY41-6551 achieves bactericidal tracheal aspirate amikacin concentrations in mechanically ventilated patients with Gram-negative pneumonia. Intensive Care Med.

[42] Chastre J, Luyt C-E (2007). Optimising the duration of antibiotic therapy for ventilator-associated pneumonia. Eur Respir Rev.

[43] Fihman V, Messika J, Hajage D, Tournier V, Gaudry S, Magdoud F, Barnaud G, Billard-Pomares T, Branger C, Dreyfuss D, Ricard JD (2015). Five-year trends for ventilator-associated pneumonia: correlation between microbiological findings and antimicrobial drug consumption. Int J Antimicrob Agents.

[44] Niederman M, Chastre J, Corkery K, Marcantonio A, Fink J, Luyt C-E, Sanchez M and The Amikacin Study Group. NKTR-061 (inhaled amikacin) reduces intravenous antibiotic use in intubated mechanically ventilated patients during treatment of Gram-negative pneumonia. ATS 18–23 May 2007, Poster A326.

[45] Colistin summary of product characteristics. https://www.medicines.org.uk/emc/medicine/27647. Accessed 25 Nov 2015.

[46] Aztreonam summary of product characteristics. https://www.medicines.org.uk/emc/medicine/22358. Accessed 25 Nov 2015.

[47] Tobramycin summary of product characteristics. https://www.medicines.org.uk/emc/medicine/19020. Accessed 25 Nov 2015.

[48] Quon B, Goss C, Ramsey B (2014). Inhaled antibiotics for lower airway infections. Ann Am Thorac Soc.

[49] Geller DE (2009). Aerosol antibiotics in cystic fibrosis. Respir Care.

[50] Zampieri F, Nassar A, Gusmao-Flores D, Taniguchi L, Torres A, Ranzani O (2015). Nebulized antibiotics for ventilator-associated pneumonia: a systematic review and meta-analysis. Crit Care.

[51] Rouby J, Bouhemad B, Monsel A, Brisson H, Arbelot C, Lu Q, the Nebulized Antibiotic Study Group (2012). Aerosolized antibiotics for ventilator-associated pneumonia: lessons from experimental studies. Anesthesiology.

[52] Kollef M, Hamilton C, Montgomery A (2013). Aerosolized antibiotics: do they add to the treatment of pneumonia?. Curr Opin Infect Dis.

[53] Dhand R (2008). Aerosol delivery during mechanical ventilation: from basic techniques to new devices. J Aerosol Med Pulm Drug Deliv.

[54] Kadrichu N, Boc S, Corkery K, Challoner P. In vitro efficiency of the Amikacin Inhale System, a novel integrated drug-device delivery system. ISICEM, 19–22 March 2013, Poster A384.

[55] Laube B, Janssens H, de Jongh F, Devadason S, Dhand R, Diot P, Everard M, Horvath I, Navalesi P, Voshaar T, Chrystyn H (2011). What the pulmonary specialist should know about the new inhalation therapies. Eur Respir J.

[56] Dhand R (2003). Maximising aerosol delivery during mechanical ventilation: go with the flow and go slow. Intensive Care Med.

[57] Miller D, Amin M, Palmer L, Shah A, Smaldone G (2003). Aerosol delivery and modern mechanical ventilation: in vitro/in vivo evaluation. Am J Respir Crit Care Med.

[58] Restrepo R, Walsh B (2012). Humidification during invasive and noninvasive mechanical ventilation: 2012. Respir Care.

[59] Lu Q, Girardi C, Zhang M, Bouhemad B, Louchahi K, Petitjean O, Wallet F, Becquemin M, Le Naour G, Marquette C, Rouby J (2010). Nebulized and intravenous colistin in experimental pneumonia caused by *Pseudomonas aeruginosa*. Intensive Care Med.

[60] Mandell LA, Wunderink RG, Anzueto A, Bartlett JG, Campbell GD, Dean NC, Dowell SF, File TM, Musher DM, Niederman MS, Torres A, Whitney CG (2007). Infectious Diseases Society of America/American Thoracic Society consensus guidelines on the management of community-acquired pneumonia in adults. Clin Infect Dis.

[61] Masterton RG, Galloway A, French G, Street M, Armstrong E, Brown E, Cleverley J, Dilworth P, Fry C, Gascoigne AD, Knox A, Nathwani D, Spencer R, Wilcox M (2008). Guidelines for the management of hospital-acquired pneumonia in the UK: report of the Working Party on hospital-acquired pneumonia of the British Society for Antimicrobial Chemotherapy. J Antimicrob Chemother.

[62] Ehrmann S, Roche-Campo F, Sferrazza Papa G, Isabey D, Brochard L, Apiou-Sbirlea G, REVA research network (2013). Aerosol therapy during mechanical ventilation: an international survey. Intensive Care Med.

[63] PARI eFlow rapid, Technical Data. http://www.pari.de/uk-en/products/lower-airways-1/eflow-rapid-nebuliser-system-1/. Accessed 13 Nov 2015.

[64] Montgomery A, Rhomberg P, Abuan T, Walters K, Flamm R (2014). Amikacin-fosfomycin at a five-to-two ratio: characterization of mutation rates in microbial strains causing ventilator-associated pneumonia and interactions with commonly used antibiotics. Antimicrob Agents Chemother.

[65] Montgomery A, Rhomberg P, Abuan T, Walters K, Flamm R (2014). Potentiation effects of amikacin and fosfomycin against selected amikacin-nonsusceptible Gram-negative respiratory tract pathogens. Antimicrob Agents Chemother.

[66] Montgomery A, Vallance S, Abuan T, Tservistas M, Davies A (2014). A randomized double-blind placebo-controlled dose-escalation Phase 1 study of aerosolized amikacin and fosfomycin delivered via the PARI Investigational eFlow^®^ Inline Nebulizer System in mechanically ventilated patients. J Aerosol Med Pulm Drug Deliv.

[67] Hallal A, Cohn S, Namias N, Habib F, Baracco G, Manning R, Crookes B, Schulman C (2007). Aerosolized tobramycin in the treatment of ventilator-associated pneumonia: a pilot study. Surg Infect (Larchmt).

[68] Kadrichu N, Corkery K, Dang T, Challoner P (2015). Performance of amikacin inhale: impact of supplemental oxygen and device orientation. Crit Care.

[69] Kadrichu N, Boc S, Corkery K and Challoner P. Influence of humidification on in vitro dose delivery for Amikacin Inhale by mechanical ventilation. ESICM, 27 September–1 October 2014, Abstract 0867.

[70] Pritchard JN (2001). The influence of lung deposition on clinical response. J Aerosol Med.

[71] Aerogen. Aeroneb^®^ Solo datasheet. http://www.aerogen.com/uploads/datasheets/Aeroneb_Solo_Product_Datasheet_FINAL_A4.pdf. Accessed 13 Nov 2015.

[72] Aerogen. Aeroneb^®^ Solo FAQs. http://www.aerogen.com/medical-community/faq/faq-aerogen-solo.html. Accessed 13 Nov 2015.

[73] Seemann S, Schmitt A, Waldner R, Hug M, Knoch M. Improving aerosol drug delivery in CF therapy. European Cystic Fibrosis Conference, 22–26 June 2005, Poster P113.

